# Reference genes for qRT-PCR normalisation in different tissues, developmental stages, and stress conditions of *Hypericum perforatum*

**DOI:** 10.7717/peerj.7133

**Published:** 2019-06-20

**Authors:** Wen Zhou, Shiqiang Wang, Lei Yang, Yan Sun, Qian Zhang, Bin Li, Bin Wang, Lin Li, Donghao Wang, Zhezhi Wang

**Affiliations:** 1 National Engineering Laboratory for Resource Development of Endangered Crude Drugs in Northwest China, Key Laboratory of the Ministry of Education for Medicinal Resources and Natural Pharmaceutical Chemistry, College of Life Sciences, Shaanxi Normal University, Xi’an, Shaanxi, China; 2 College of Chemistry, Biology and Materials Science, East China University of Technology, NanChang, China

**Keywords:** Reference genes, *Hypericum perforatum L.*, qRT-PCR, Normalization, Gene expression

## Abstract

*Hypericum perforatum L*. is a widely known medicinal herb used mostly as a remedy for depression because it contains high levels of naphthodianthrones, phloroglucinols, alkaloids, and some other secondary metabolites. Quantitative real-time PCR (qRT-PCR) is an optimized method for the efficient and reliable quantification of gene expression studies. In general, reference genes are used in qRT-PCR analysis because of their known or suspected housekeeping roles. However, their expression level cannot be assumed to remain stable under all possible experimental conditions. Thus, the identification of high quality reference genes is essential for the interpretation of qRT-PCR data. In this study, we investigated the expression of 14 candidate genes, including nine housekeeping genes (HKGs) (*ACT2*, *ACT3*, *ACT7*, *CYP1*, *EF1*-α, *GAPDH*, *TUB*-α, *TUB*-β, and *UBC2*) and five potential candidate genes (*GSA*, *PKS1*, *PP2A*, *RPL13*, and *SAND*). Three programs—GeNorm, NormFinder, and BestKeeper—were applied to evaluate the gene expression stability across four different plant tissues, four developmental stages and a set of abiotic stress and hormonal treatments. Integrating all of the algorithms and evaluations revealed that *ACT2* and *TUB*-β were the most stable combination in different developmental stages samples and all of the experimental samples. *ACT2*, *TUB*-β, and *EF1*-α were identified as the three most applicable reference genes in different tissues and stress-treated samples. The majority of the conventional HKGs performed better than the potential reference genes. The obtained results will aid in improving the credibility of the standardization and quantification of transcription levels in future expression studies on *H. perforatum*.

## Introduction

An increasing number of studies on the gene expression levels in plants are being carried out to better understand the signaling and metabolic pathways underlying the developmental processes involved in plant development and growth, as well as plant responses to biotic and abiotic stresses (Moreno-Risueno et al., 2010; Yamaguchi et al., 2010; Wang et al., 2011). Methods for assessing gene expression include northern blot, gene chips, semi-PCR, RNase protection analysis, and Quantitative real-time PCR (qRT-PCR). qRT-PCR has become a very popular and effective method for detecting and quantifying gene transcription levels because of its high sensitivity, specificity, reproducibility, and accuracy (Nolan, Hands & Bustin, 2006). Reliable quantification of gene expression levels using qRT-PCR analysis requires the standardization and fine-tuning of several parameters, such as the amount of initial sample, RNA recovery and integrity, enzymatic efficiency of cDNA synthesis and PCR amplification, and the overall transcriptional activity of the tissues or cells analyzed (Nolan, Hands & Bustin, 2006; Udvardi & Scheible, 2008). The expression stability of frequently used reference genes also cannot be neglected. Therefore, the normalization of the transcript levels of test genes is essential for minimizing technical differences that arise in different samples and experimental conditions (Udvardi & Scheible, 2008). More importantly, the selection of stable reference genes must be done before qRT-PCR analysis.

An appropriate reference gene is not affected by the experimental conditions and remains at an invariable level among samples (Bustin & Mueller, 2005). However, the transcription levels of the traditional reference genes and even some housekeeping genes (HKGs) can vary between different types of tissue and under different treatment conditions (Leal et al., 2015). Therefore, a growing number of studies have been published on the analysis and evaluation of the stability of internal reference genes in plant tissues under different conditions. Consequently, different reference genes should be applied for different experimental materials and conditions. In addition, the use of reference genes with lower stability may lead to an erroneous understanding of the qRT-PCR results and mask the true nature of gene expression (Brattelid & Levy, 2011; Bustin & Nolan, 2004).

*Hypericum perforatum L*. (commonly known as St. John’s wort) is a widely known medicinal herb used mostly as a remedy for depression (Veronika, 2003). Pure essential compounds isolated from *H. perforatum*, namely naphthodianthrones and phloroglucinols, have been shown to possess anti-depressive, anti-cancer, anti-viral, anti-inflammatory, and other activities (Birt et al., 2009; Filippo et al., 2011). Xanthones and flavonoids have also been identified in extracts from this plan (Schröder, 1997). To date, a limited number of qRT-PCR studies focusing on *H. perforatum* have been published. Velada et al. (2014) studied the stability of 11 candidate reference genes analyzed in *H. perforatum* plants subjected to only cold and heat stresses, and *TUB* was found to be the most stable gene under both experimental conditions. Therefore, it is essential to screen suitable reference genes in different tissues of *H. perforatum* under different experimental conditions. Ribosomal RNA and some HKGs are usually used as reference genes, such as actin (*ACT*), tubulin (*TUB)*, glyceraldehyde-3-phosphate dehydrogenase (*GAPDH*), and polyubiquitin (*UBQ*) (Costa et al., 2015; Goulao, Fortunato & Ramalho, 2012; Llanos, François & Parrou, 2015; Willems, Leyns & Vandesompele, 2008), whereas many studies have revealed that the most commonly used HKGs are not always reliable among different experimental samples. Thus, an evaluation to screen the optimal HKGs in different species is urgently needed (Ohl et al., 2005; Selvey et al., 2001; Thellin et al., 1999).

This study aimed to assess the expression stabilities of 14 reference genes in 15 experimental samples using qRT-PCR, including nine traditional HKGs and five potential reference genes: *GAPDH*, actin (*ACT2*, *ACT3*, and *ACT7*), ubiquitin-conjugating (*UBC2*), elongation factor (*EF1-a*), tubulin (*TUB*-α and *TUB*-β), cyclophilin (*CYP1*), polyketide synthase (*PKS1*), glutamate semialdehyde aminomutase (*GSA*), SAND family protein (*SAND*), ribosomal protein L (*RPL13*) and protein phosphatase 2A (*PP2A*). These genes were selected from the *H. perforatum* genome sequencing data obtained in our lab. In this study, we evaluated the transcriptional stability of these genes in different tissues, developmental stages and under different stress conditions to determine the most stable reference genes.

## Materials and Methods

### Plant materials

*H. perforatum* seeds (2*n* = 2× = 16) were germinated on a seedling bed in the glasshouse (25 ± 2 °C, natural lighting, 60–80% humidity). Whole plant tissues were collected at the one-month-old (1M), two-month-old (2M), three-month-old (3M), and six-month-old (6M) stages. Samples of different tissues (leaf, flower, stem, and root) were taken from two-year-old plants (2*n* = 2× = 16). 3M seedlings were subjected to abiotic stresses and hormonal treatments, including 10 μM salicylic acid (SA), 200 μM methyl jasmonate (MeJA), 100 μM abscisic acid (ABA), one mM AgNO_3_ (Ag), 200 μM CuSO_4_ (Cu), 100 mM NaCl (Na), low temperature (4 °C) and wounding (W). The stress-treated samples were each collected 6 h after the corresponding treatments, and the control groups were collected following non-treatment. All samples were collected in three replicates, frozen in liquid nitrogen immediately, and then stored at −80 °C.

### Total RNA isolation and cDNA synthesis

Total RNA was extracted using the Polysaccharide and Polyphenols Plant Quick RNA Isolation Kit (centrifugal column type; Waryong, Beijing, China). The genomic DNA was digested with RNase-free DNase I (TaKaRa, Kusatsu, Japan). The total RNA was quantified using the absorbance at A_260_/A_280_ and A_260_/A_230_ nm measured with a NanoDrop 2000c spectrophotometer (Thermo Scientific, Waltham, MA, USA). A 1% (p/v) agarose gel was run to visualize the integrity of the RNA. Only RNA samples with an A_260_/A_280_ wavelength ratio between 1.9 and 2.1 and an A_260_/A_230_ ratio close to 2.0 were used for cDNA synthesis. Then, 1.0 μg DNA-free total RNA was used to synthesize first-strand cDNAs with a PrimeScript RT Reagent Kit (TaKaRa, Kusatsu, China) in a 20 μL volume. All cDNA samples were diluted (1:40) with DNase/RNase-free deionized water for qRT-PCR.

### Selection of reference genes and primer design

We performed genomic sequencing of *H. perforatum* using Illumina paired-end, 10× Genomics linked reads and PacBio SMART (GenBank accession numbers: MK054303, MK106356–MK106365). The transcriptome sequencing of *H. perforatum* for the roots, stems, leaves, and flowers assisted annotation. The 14 candidate genes, including nine traditional HKGs (*ACT2*, *ACT3*, *ACT7*, *CYP1*, *EF1*-α, *GAPDH*, *TUB*-α, *TUB*-β, and *UBC2*) and five potential reference genes (*PKS1*, *GSA*, *RPL13*, *SAND*, and *PP2A*) which have been used as candidate genes in other studies (He et al., 2016; Li et al., 2017; Velada et al., 2014), were selected for the assessment of the most stably expressed reference genes (Table S1). To ensure the accuracy of the reference gene predictions, we first screened the candidate genes according to the genome annotation of each, which was assigned based on the best match of the alignments using Blastp to SwissProt, KEGG, NR, and TrEMBL databases. Then the coding sequences of the 14 selected genes were used as queries for BLAST orderly through the TAIR database (http://www.arabidopsis.org/) to further ensuring accuracy. The sequences with the highest homology with Arabidopsis are shown in Table 1. The primers of all the genes were designed using GenScript (https://www.genscript.com) with a melting temperature between 59 and 61 °C, a primer length of 20–25 bp, and an amplicon length of 70–180 bp. The descriptions of the candidate reference genes, primer sequences, and qRT-PCR amplification efficiencies are presented in Table 1.

**Table 1:** Candidate genes, primers, and different parameters derived from the qRT-PCR analysis in *H. perforatum*.

Gene symbol	Gene name	Accession No.	Amplicon size (bp)	Primer sequence U/L (5′–3′)	Tm (°C)	Efficiency (%)
*ACT2*	Actin 2	MK054303	101	Fw: AGGAGTCCCTCCACGACCAC	83.6	97.6
				Rv: GCCGTTGTGTACCGGGTAGG		
*ACT3*	Actin 3	MK106364	139	Fw: ATCCTTCCCACGGTGGTTGC	88.2	102.9
				Rv: CAATCGCCTCGTCGCCTACA		
*ACT7*	Actin 7	MK106365	131	Fw: GAGCAGCAGCAGGTCGACAA	83.0	107.6
				Rv: ACCCACTCGAGCCCAGTGTA		
*CYP1*	Cyclophilin	MK106359	163	Fw: AGGGATCCAGCTTCCACCGT	87.8	96.2
				Rv: GCGTTGGCCATGGAGAGGAT		
*EF1*-α	Elongation factor 1-alpha	MK106356	121	Fw: TGGAGGCTCTCCCTGGTGAC	85.8	105.6
				Rv: AAGTTGGCAGCCTCCTTGGC		
*GAPDH*	Glyceraldehyde-3-phosphate dehydrogenase A subunit	EU301783	76	Fw: AGGCCTCCCACCTCCTCAAG	84.7	105.1
				Rv: GGTTGACAGGGTTGCGGTCA		
*TUB*-α	Alpha tubulin	MK106362	132	Fw: TGCTGCGGTTGCCACCATTA	84.8	109.5
				Rv: CGCTGCACCTTTGCAAGATCG		
*TUB*-β	Beta tubulin	MK106361	170	Fw: CGACGGGAGTGACAGCCTTG	86.5	108.6
				Rv: CGACCACATCGCTCGTCTCC		
*UBC2*	Ubiquitin-conjugating enzyme 2	MK106357	103	Fw: AGGAGGAGGCGCCTTTGAGA	84.5	98.2
				Rv: CGCAAGAGCCGGTCCATTCA		
*GSA*	Glutamate-1-semialdehyde 2,1-aminomutase	KJ624985	78	Fw: GGTGTCAGGATGGCGGTGTC	85	94.4
				Rv: GGGCTGGCCACCAACTGATT		
*PKS1*	Polyketide synthase 1	EF186675	76	Fw: ACGGACGCTGCCATCAA	82.5	98.3
				Rv:ACATAACCGTGTACCTTGTC		
*PP2A*	Protein phosphatase 2A	MK106360	93	Fw: GGCAAGTGCCCAGACACCAA	81.2	95.5
				Rv: CAGCGCCACTAGCAGCGTAA		
*RPL13*	Ribosomal protein L	MK106363	151	Fw: CAGCGCTGGATGTTCGAGGA	84.5	92.5
				Rv: TGGTGGAAGCCAACCTCCCA		
*SAND*	SAND family protein	MK106358	111	Fw: CTCCCTCGCACTGGGACAAC	83.2	97.1
				Rv: AAGACCAGCAGGACCACCCA		

### qRT-PCR conditions and analysis

PCR reactions were performed on the Roche LightCycler 96 system using SYBR^®^ Master Mix. Reactions were performed in triplicate in 20 μL volumes containing five μL 30-fold diluted synthesized cDNA, 10 μL SYBR^®^ Master Mix, 0.4 μL 10 mM forward primer, 0.4 μL 10 mM reverse primer and 4.2 μL DNase/RNase-free deionized water. The cycling conditions were 95 °C for 30 s, 45 cycles of 95 °C for 5 s, and 60 °C for 30 s, and a final melting curve analysis. Each reaction was set up with a negative control group and triple technical replicates were performed in each of three biological samples. To calculate the amplification efficiency from 10-fold continuous dilutions of the cDNA (10^0^, 10^−1^, 10^−2^, and 10^−3^) for each gene, standard curves were constructed to obtain the correlation coefficients (*R*^2^) and slope values. Using these standard curves, the corresponding PCR amplification efficiencies (*E*) were calculated (*E* = (10^−1/slope^ −1) × 100) (Aleksandar et al., 2004).

### Assessment of expression stability

The expression stability of the genes was analyzed using three different Visual Basic applets, GeNorm (Schlotter et al., 2009), NormFinder (Obrero et al., 2011; Zhang et al., 2016), and BestKeeper (Rhinn et al., 2008; Rotenberg et al., 2006). GeNorm derives a stability measure (*M*-value) via the stepwise exclusion of the least stable gene, and creates a stability ranking (Hu et al., 2009). Genes with *M* < 1.5 are generally considered stable reference genes (Tong et al., 2009). This measure is based on the principle that the expression ratio of two ideal control genes should be identical in all samples; thus, genes with the lowest *M*-value are the most stably expressed (Dekkers et al., 2012). The GeNorm pairwise variation (V) values between ranked genes (*V*_n_/*V*_n+1_) were determined for the optimal number of reference genes to be used in the quantitative analysis experiments. A cut-off of 0.15 (*V*_n_ value) is usually applied (Vandesompele et al., 2002). NormFinder uses an ANOVA-based model to estimate intra- and intergroup variation within tissues or treatments (“groups” in NormFinder terminology) to assess the expression stability (Marchal et al., 2013). For GeNorm and NormFinder, the raw Ct values need to be converted to relative quantities (Q) using the formula Q = 2^−^^ΔCt^, in which ΔCt = each average Ct value−minimum Ct value (Yang et al., 2015). In BestKeeper, the coefficient of variance (CV) and the standard deviation (SD) were calculated using the Ct values, with lower CV and SD values indicating higher stability (Migocka & Papierniak, 2011). The mean standard error and level of statistical significance were calculated using GraphPad Prism 6.0, and the level of statistical significance was assessed using ^∗^*P* < 0.05, ^∗∗^*P* < 0.01, and ^∗∗∗^*P* < 0.001.

### Validation of reference gene stability

The relative expression of the *HpHYP1* gene in different tissues was measured and standardized by using the most stable and unstable candidate genes as internal references to verify the reliability of the selected genes according to the 2^−ΔΔCt^ method (Kumar et al., 2011). Three technical replicates were performed for each biological sample.

## Results

### Primer specificity and expression level analysis of candidate reference genes

**Figure 1:**
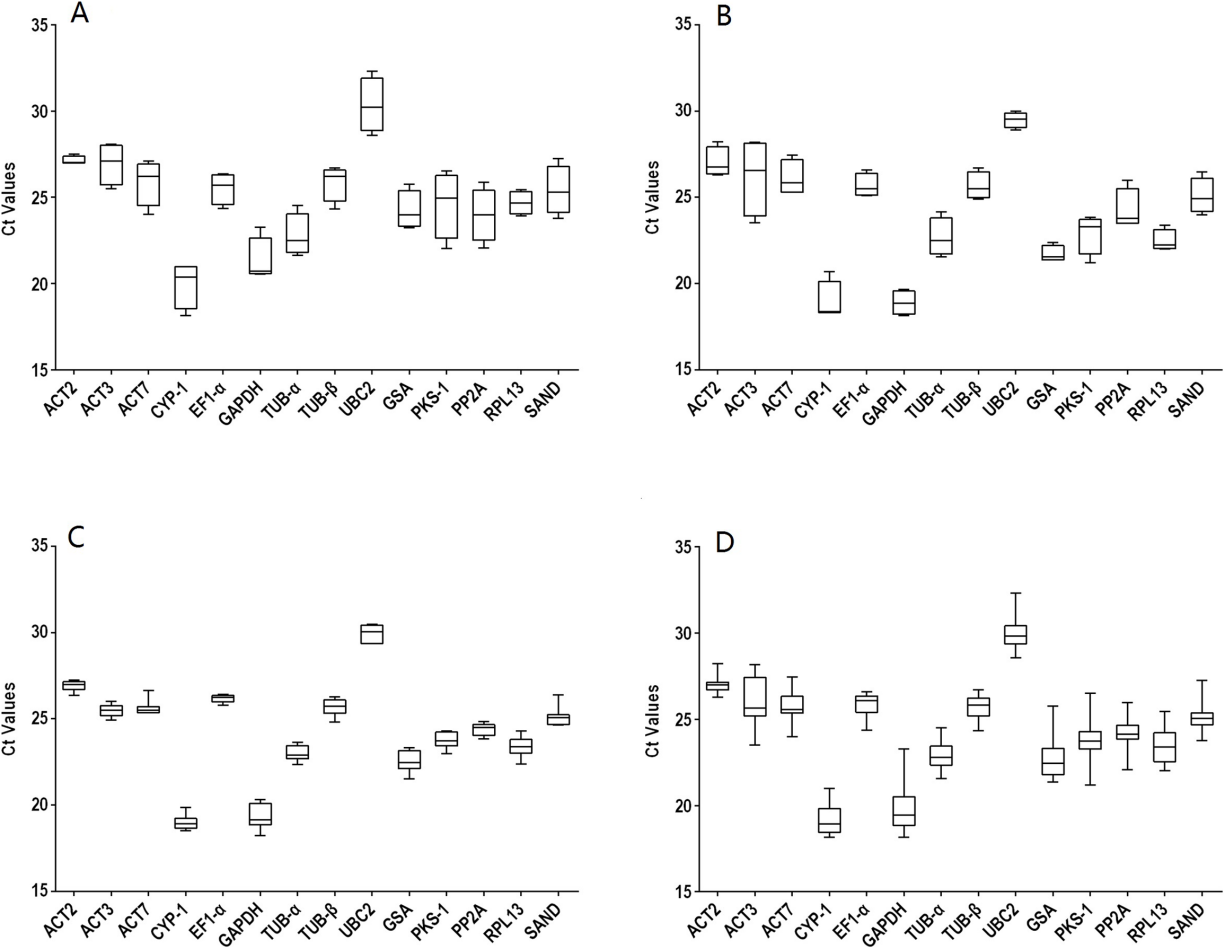
Distribution overview of the expression profiles of 14 candidate genes. (A) different tissues, (B) developmental stage seedlings, (C) stress-treated seedlings, (D) data from all experimental conditions combined. The line across the box is the median. The box indicates the 25th and 75th percentiles; whisker caps indicate the maximum and minimum values; and dots indicate outliers.

The gene names and abbreviations, accession numbers, primer sequences, amplification efficiencies, amplicon sizes, Tm values, and molecular functions are listed in Table 1 and Table S1. The amplification efficiencies listed in Table S2 ranged from 92.5% (*RPL13*) to 109.5% (*TUB*-α), the Tm values varied from 81.2 °C (*PP2A*) to 87.8 °C (*CYP1*), and the amplicon sizes were between 76 (*GAPDH* and *PKS1*) and 170 bp (*TUB*-β). Furthermore, primer specificities were determined using melting curves (Fig. S1) and triple technical replicates were performed for each of the three biological samples. A single band indicated the correct size of each pair. The average raw CT values of different genes ranged from 18.15 to 32.32 (Table S3). Data were analyzed within experiments and divided into four groups: tissues from two-year-old plants (TS: R, S, L, and F), seedling developmental stages (SG: 1M, 2M, 3M, and 6M), 3M seedlings exposed to abiotic stresses (ST: SA, MeJA, ABA, Cu, Ag, Na, 4 °C and W) and a combination of all experimental conditions (TT). The expression levels of the 14 selected genes in the four groups are shown in Fig. 1. Among them, *UBC2* (TS) had the highest Ct value, but *GAPDH* (SG) had the lowest, indicating their levels of expression.

### Stability of candidate reference genes

**Figure 2:**
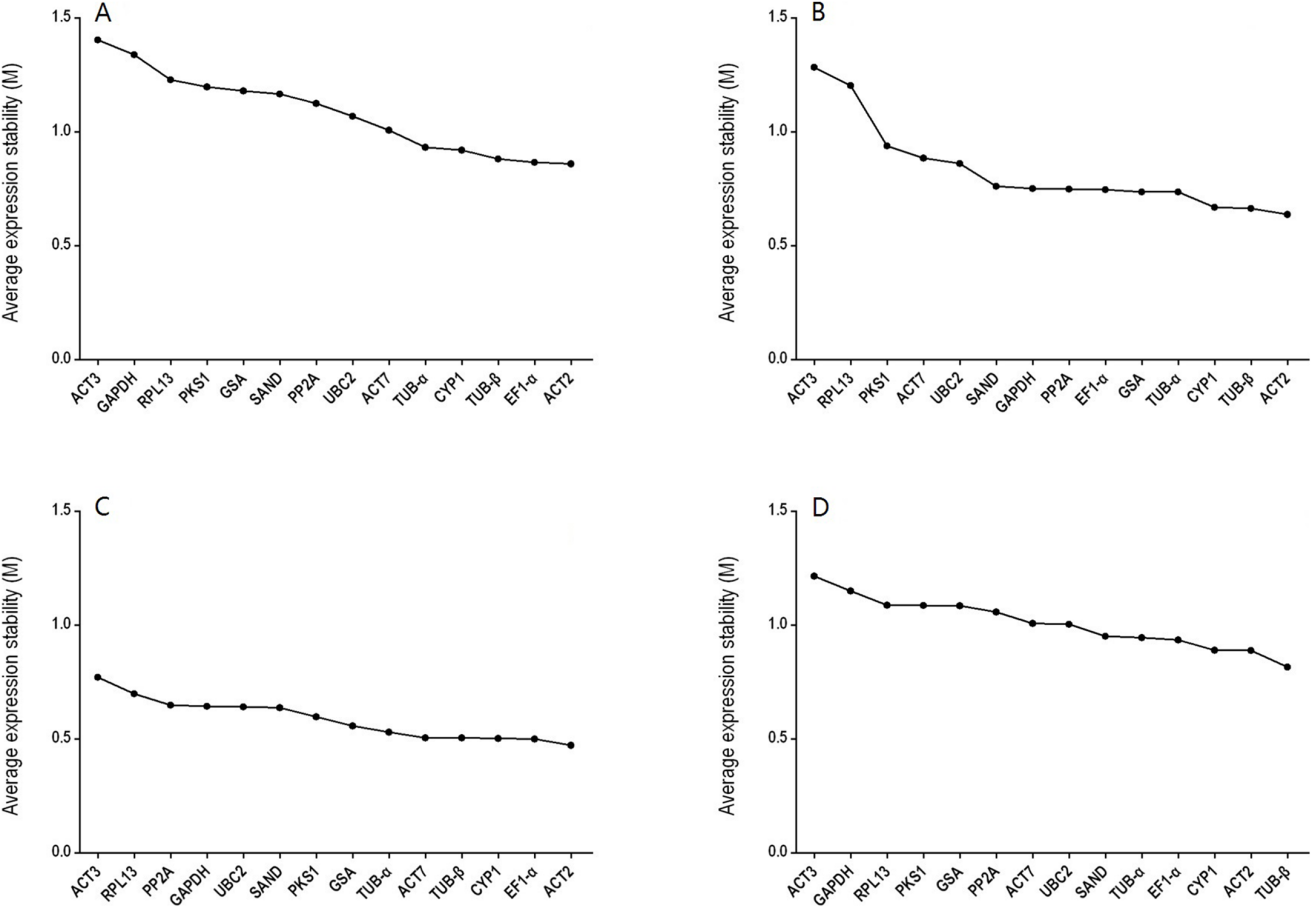
Average expression stability (*M*-value) of the 14 candidate reference genes calculated using GeNorm. (A) different tissues, (B) developmental stage seedlings, (C) stress-treated seedlings, and (D) data from all experimental groups combined. Lower average expression stability (*M*-value) indicates more stable expression.

**Figure 3:**
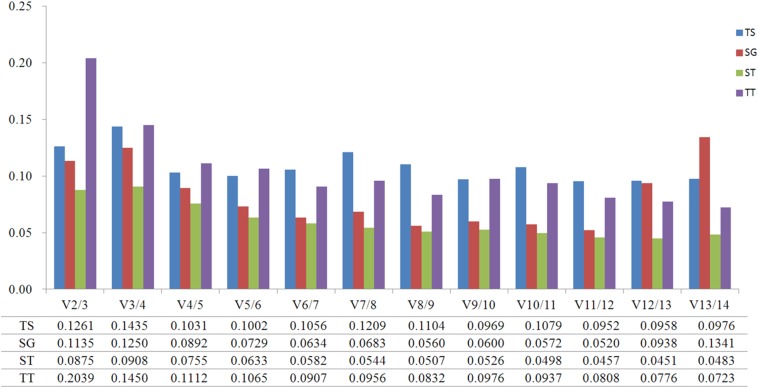
Pairwise variation (V) calculated using GeNorm to determine the optimal number of reference genes. (TS) different tissues, (SG) developmental stage seedlings, (ST) stress-treated seedlings, and (TT) data from all experimental groups combined. The average pairwise variations *V*_n_/*V*_n+1_ were analyzed between the normalization factors NF_n_ and NF_n+1_ to indicate the optimal number of reference genes in different samples.

GeNorm was used to calculate the normalization factor from the geometric mean of the genes to identify the most stably expressed gene. The *M*-value is defined as the average pairwise variation of a particular gene with all other potential reference genes. The expression stability ranking of the 14 reference genes in the TT samples was arranged as follows: *TUB*-β > *ACT2* > *CYP1* > *EF1*-α > *TUB*-α > *SAND* > *UBC2* > *ACT7* > *PP2A* > *GSA* > *PKS1* > *RPL13* > *GAPDH* > *ACT3*. Except for in the TT group, *ACT2* and *ACT3* were the genes with the lowest and highest *M*-values, respectively, (Fig. 2; Table S4). The *V*_2/3_ values for almost all of the experimental sets in *H. perforatum* were lower than the cut-off threshold of 0.15 (Fig. 3), which indicated that the combination of two reference genes (*ACT2* and *TUB*-β, or *ACT2* and *EF1-a*) can accurately standardize these samples. For the TT group, *V*_2/3_ was 0.2039, which indicated that the top three reference genes (*ACT2*, *TUB*-β, and *CYP1*) were required for accurate normalization.

NormFinder with lower values indicating higher stability is another Excel application that ranks the candidate genes based on their minimal combined inter- and intragroup variation of expression. The results (Table 2) indicated that *ACT2* was the most stable gene in the SG and ST groups. *EF1*-α and *TUB*-β ranked first in the TS and TT groups respectively. *ACT3* was at the end of the rankings in all groups calculated using NormFinder. The stability order of the candidate genes calculated using NormFinder was nearly the same as that calculated using GeNorm, although there was a small difference. For example, *ACT2* was identified as the most stable reference gene in the TS group with the GeNorm analysis, while its stability rankings placed it third within the NormFinder analysis.

**Table 2:** Ranking and expression stability values of the candidate reference genes calculated by NormFinder.

Rank	TS		SG		ST		TT	
	Gene	Stability value	Gene	Stability value	Gene	Stability value	Gene	Stability value
1	*EF1*-α	0.090	*ACT2*	0.048	*ACT2*	0.141	*TUB*-β	0.246
2	*TUB*-β	0.248	*TUB*-β	0.048	*ACT7*	0.179	*ACT2*	0.332
3	*ACT2*	0.323	*TUB*-α	0.190	*EF1*-α	0.194	*CYP1*	0.346
4	*CYP1*	0.371	*EF1*-α	0.205	*TUB*	0.195	*EF1*-α	0.398
5	*ACT7*	0.462	*CYP1*	0.251	*CYP1*	0.205	*TUB*-α	0.445
6	*UBC2*	0.521	*GSA*	0.304	*TUA*	0.247	*SAND*	0.453
7	*GSA*	0.620	*GAPDH*	0.308	*GSA*	0.262	*ACT7*	0.511
8	*PP2A*	0.644	*PP2A*	0.325	*PKS1*	0.311	*UBC2*	0.532
9	*GAPDH*	0.653	*SAND*	0.337	*SAND*	0.335	*PKS1*	0.562
10	*PKS1*	0.663	*UBC2*	0.487	*UBC2*	0.341	*PP2A*	0.586
11	*SAND*	0.687	*RPL13*	0.491	*RPL13*	0.353	*RPL13*	0.594
12	*TUB*-α	0.710	*ACT7*	0.540	*GAPDH*	0.375	*GSA*	0.602
13	*RPL13*	0.789	*PKS1*	0.771	*PP2A*	0.401	*GAPDH*	0.668
14	*ACT3*	0.934	*ACT3*	1.297	*ACT3*	0.462	*ACT3*	0.688

**Note:**

(TS) Different tissues, (SG) developmental stage seedlings, (ST) stress-treated seedlings, and (TT) data from all experimental conditions combined.

BestKeeper is another Excel-based program and is able to compare the expression levels of up to 10 target genes, each in up to 100 biological samples. The CV and SD values of the 14 candidate genes computed using BestKeeper are represented in Table 3. The smaller the SD and the CV values are, the better the stability of the internal reference genes. The expression of the reference gene was unstable when SD value is greater than one. The results revealed that *ACT2* was the most stable genes for the ST and TT samples, while *TUB*-β ranked first in the TS and SG samples.

**Table 3:** Ranking of the candidate reference genes and their expression stability calculated by BestKeeper.

Rank	TS			SG			ST			TT		
	Gene	SD	CV	Gene	SD	CV	Gene	SD	CV	Gene	SD	CV
1	*TUB*-β	0.184	0.677	*TUB*-β	0.294	0.996	*ACT2*	0.181	0.692	*ACT2*	0.303	1.121
2	*RPL13*	0.520	2.106	*ACT2*	0.329	1.514	*EF1*-α	0.231	0.857	*TUB*-β	0.551	2.145
3	*CYP-1*	0.766	2.963	*GSA*	0.454	2.020	*CYP-1*	0.274	1.070	*CYP1*	0.597	2.308
4	*TUB*-α	0.798	3.291	*RPL13*	0.585	3.098	*TUB*-β	0.281	1.103	*TUB*-α	0.606	2.650
5	*SAND*	0.870	3.819	*GAPDH*	0.610	2.258	*RPL13*	0.285	1.167	*RPL13*	0.610	2.423
6	*ACT7*	0.925	3.640	*EF1*-α	0.610	2.378	*PKS-1*	0.319	1.678	*EF1*-α	0.628	2.099
7	*ACT2*	0.943	3.641	*SAND*	0.695	2.771	*TUB*-α	0.356	1.499	*ACT7*	0.633	2.452
8	*EF1*-α	0.958	3.787	*CYP1*	0.793	3.036	*SAND*	0.357	1.550	*PP2A*	0.644	2.655
9	*GAPDH*	0.976	4.580	*TUB*-α	0.803	3.540	*ACT3*	0.362	1.440	*SAND*	0.763	3.248
10	*ACT3*	0.985	3.656	*UBC2*	0.833	3.211	*PP2A*	0.378	1.471	*UBC2*	0.770	4.005
11	*PP2A*	0.995	4.151	*PKS-1*	0.855	3.732	*ACT7*	0.399	1.705	*PKS1*	0.831	3.498
12	*GSA*	1.013	5.071	*PP2A*	0.859	3.539	*UBC2*	0.436	1.457	*GSA*	0.893	3.928
13	*UBC2*	1.165	3.841	*ACT7*	0.873	4.605	*GSA*	0.471	2.090	*GAPDH*	0.947	4.803
14	*PKS-1*	1.393	5.655	*ACT3*	1.888	7.201	*GAPDH*	0.562	2.909	*ACT3*	1.039	3.989

**Note:**

(TS) Different tissues, (SG) developmental stage seedlings, (ST) stress-treated seedlings, and (TT) data from all experimental conditions combined.

### Reference gene validation

**Figure 4:**
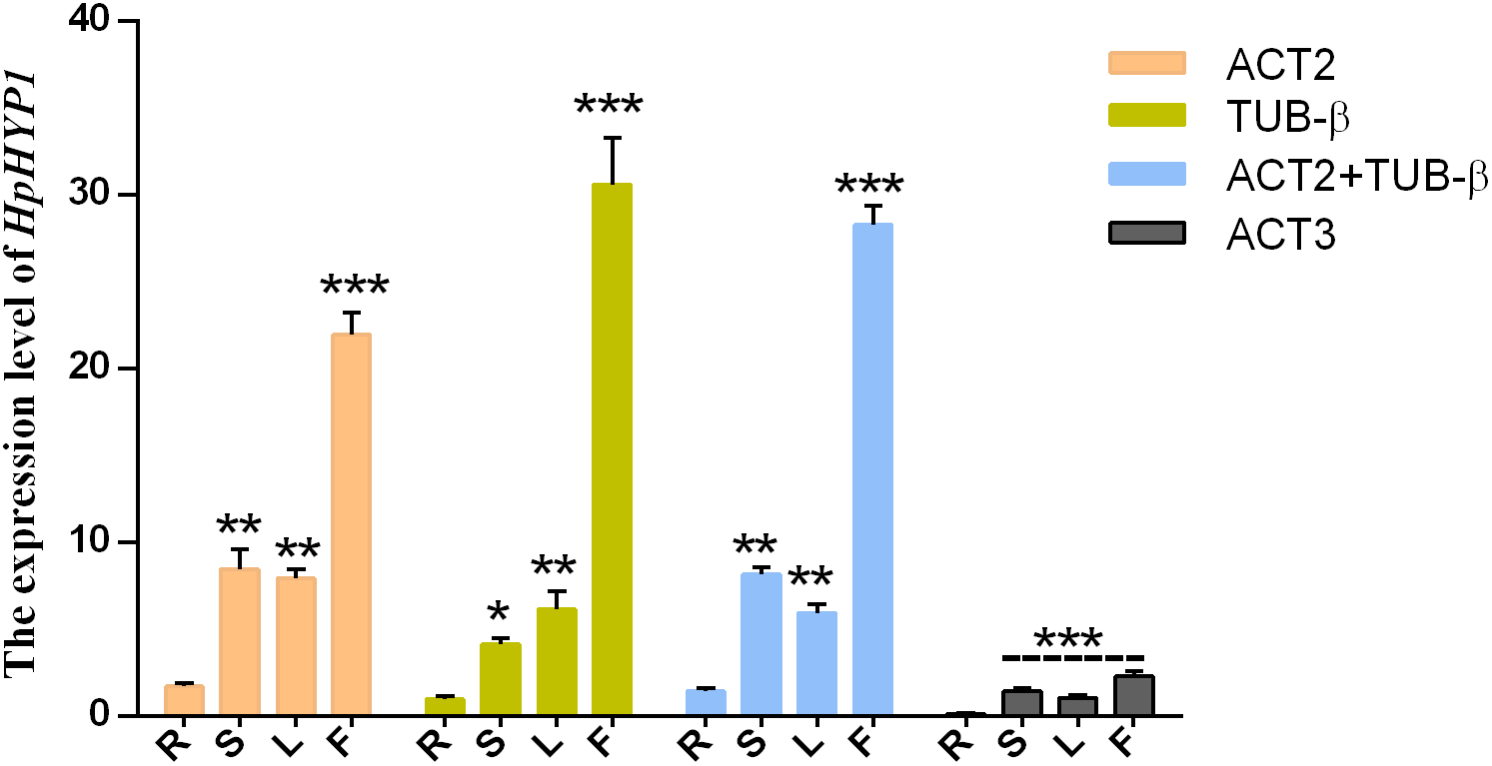
Relative expression levels of *HpHYP1* in (R) root, (S) stem, (L) leaf, and (F) flower used for normalization using the most stable reference genes or a combination and the least stable genes. Error bars indicate the mean standard error calculated from three biological replicates. The statistical level was according to ^∗^*P* < 0.05, ^∗∗^*P* < 0.01, and ^∗∗∗^*P* < 0.001.

To test the reliability of the results, the relative expression patterns of the target gene *HpHYP1*, which belongs to the PR-10 family and is associated with stress control, were evaluated using different internal control genes in the roots, stems, leaves, and flowers. Based on the results of the present study, the top two stable genes (*ACT2* and *TUB*-β) and the most unstable gene (*ACT3*) were chosen as internal controls. As shown in Fig. 4, when normalized using *ACT2* as the reference genes, the transcript abundance of *HpHYP1* was upregulated compared with the results in the root samples. When *ACT2* and *TUB*-β (as identified using GeNorm) were used as the internal references, the expression patterns were similar to those obtain with *ACT2*. When normalization was based on *TUB*-β alone, the expression level of *HpHYP1* was still up-regulated. The only difference was that the expression level in stems was lower than that in leaves, although the difference was not significant. In general, when stable reference genes, including *ACT2*, *TUB*-β, and their combination, were used as internal parameters, *HpHYP1* had the highest expression level in flowers. When normalized using the less stable gene *ACT3*, the target genes in all tissues were expressed at lower levels but showed significantly up-regulation (*P* < 0.001). Thus, the selection of inappropriate reference genes can lead to over- or under-estimation of the relative transcript level, which might lead to a biased result.

## Discussion

The reliability of qRT-PCR data will be greatly improved by the inclusion of a reference gene with a transcription level that is invariable across different experimental conditions. Numerous published studies have verified some common reference genes, especially the HKGs, in many species, such as in *Arabidopsis thaliana* (Dekkers et al., 2012), *Salvia miltiorrhiza* (Espinosa et al., 2015), *Cucumis sativus* (Yi et al., 2004), *Gentiana macrophylla* (Aleksandar et al., 2004), and *Isatis indigotica* (Li et al., 2017). In *G. macrophylla* (He et al., 2016), *SAND1* and *EF1*-α*4* were found to be the most suitable overall; *GAPC2* and *SAND1* were identified as the best reference genes for the roots under abiotic stresses, while *SAND1* and *EF1*-α*4* were found to be the best for stressed leaves. For the tung tree, *ACT7*, *UBQ*, *GAPDH*, and *EF1*-α were the four optimal reference genes in all samples and developing seeds. *ACT7*, *EF1*-β, *GAPDH*, and *TEF1* were the top four candidate genes for different tissues. However, the appropriate reference genes for *H. perforatum* under different experimental conditions had not yet been identified, possibly because there is very limited genomic information for *H. perforatum* in the NCBI database. With the rapid development of whole-genome sequencing technology, deciphering the genomes of medicinal herbs is a vital step in understanding and improving their medicinal value. For that reason, we assembled the first high-quality sequence of the *H. perforatum* genome. Therefore, the reported literature on *H. perforatum* (Velada et al., 2014; Yao, 2011) combined with the obtained genomic data provided the foundation for selecting reference genes. Additionally, the *HpHYP1* gene, belonging to the PR-10 family associated with stress control, was used for validation of the candidate reference genes.

Housekeeping genes like those that participated in cell structure maintenance (*ACT* and *TUB*) or basic cellular processes (*UBC* and *CYP*) remain widely applied, but their expression can vary between different types of tissue (normal and pathological samples) and under different treatment conditions (drugs and chemicals) (Thorrez et al., 2008; Robinson, Sutherland & Sutherland, 2007). Thus, normalization with multiple reference genes is becoming a popular and standard practice in plant research (Pfaffl et al., 2004). In this work, 14 reference genes were chosen, including nine traditional HKGs and five potential reference genes. We assessed the expression patterns of these genes in different tissues, in different developmental stages, and under various abiotic stress treatments to identify the most stable HKGs for qRT-PCR analyses. As shown in Fig. 2, no one gene had a constant CT value, demonstrating how important it is to identify the most suitable reference gene for normalizing expression under all detection conditions in *H. perforatum*.

The analytical procedures applied in our research based on statistical algorithms to assess the stability of reference genes are commonly used by researchers to select the best reference genes (Haller et al., 2004; Jarošová & Kundu, 2010; De Kok et al., 2005; Szabo et al., 2004; Vandesompele et al., 2002). The three algorithms produced different results for the ranking of the 14 candidate reference genes, indicating the importance of using multiple types of software to obtain the best results. Furthermore, a comparison of the results of different algorithms used to assess reference genes results in a better evaluation and reduces the risk of the artificial selection of co-regulated transcripts (Ayers et al., 2007). NormFinder and GeNorm use almost the same arithmetic, but GeNorm is used to determine not only the most stable reference genes but also the optimal number of gene combinations. In the present study, when the data were combined to determine the optimal number of reference genes, the pairwise variation of *V*_2/3_ values for all of the experimental sets was lower than the cut- off threshold of 0.15 except for the TT group (Fig. 4). Thus, these results indicate that the best combination (*ACT2* and *TUB*-β, or *ACT2* and *EF1-a*) should be used to improve the accuracy of the quantitative expression analysis of *H. perforatum*. For the TT group, *V*_2/3_ was higher than 0.15, which indicated that the top three reference genes (*ACT2*, *TUB*-β, and *CYP1*) were required for accurate normalization.

In our research, GeNorm and NormFinder produced similar rankings for stability values, while BestKeeper always produced different rankings. Specifically, *ACT2* was identified as the most stable gene for the SG and ST groups, and *TUB*-β was the most stable gene in the TT group according to NormFinder and GeNorm. The difference was that *TUB*-β was ranked first in the TS and SG groups and that *ACT2* was ranked first in the ST and TT groups by BestKeeper. *RPL13* was given a relative top ranking in all groups. In contrast, the expression stability value of *RPL13* according to GeNorm and NormFinder was very low. Other previous studies have also reported similar differences between BestKeeper and other methods (Rapacz, Stępień & Skorupa, 2012). This variation in the results may be explained by the difference in the algorithms implemented by the three software packages. Homologous genes are widely used as reference genes for gene expression analysis. For example, *ACT6*, *ACT8*, and *ACT7* were selected as internal controls for stress treatments in *Fortunella crassifolia* (Pfaffl et al., 2004); *UBC19*, *UBC22*, and *UBC29* were selected as reference genes in the context of the relevant experimental conditions in *I. indigotica* (Li et al., 2017); and *EF1A2a*, *EF1A2b*, and *EF1A1a1* were identified as the best reference genes under all tested conditions in *Glycine max* (Raman et al., 2015). Nevertheless, as can be seen in our results, the three homologous genes (*ACT2*, *ACT3*, and *ACT7*) exhibited totally different expression levels when used for the normalization of qRT-PCR, especially *ACT2* and *ACT3* (Tables 2–3; Fig. 3). Therefore, although the paralogous genes have similar structures, their expression levels are entirely different in terms of gene expression quantification.

In summary, our results indicate that the selection of reference genes has a significant impact on the normalized gene expression data in qRT-PCR experiments. We investigated the expression of 14 candidate reference genes across a large number of *H. perforatum* samples to identify the most stable genes for normalizing gene expression. The results of this study will provide useful information for future genomics and transcriptomics studies on this valuable medicinal plant.

## Conclusion

Based on the gene stability analysis, we identified that *ACT2* and *TUB*-β were the most stable combination in different developmental stages samples and all of the experimental samples. *ACT2*, *TUB*-β, and *EF1*-α were considered to be the three most applicable reference genes in different tissues and stress-treated samples. In conclusion, the reference genes identified in this study provides basic background information for qRT-PCR studies in *H. perforatum*. It will contribute to accurate and consistent expression analysis for functional genomic research.

## Supplemental Information

10.7717/peerj.7133/supp-1 Supplemental Information 1 Primer specificity. Melting curves for the fourteen candidate genes show single peaks. For each sub-graph, temperature is displayed in the x axis, the derivative reporter signal is displayed in the y axis.

10.7717/peerj.7133/supp-2 Supplemental Information 2 The coding sequences of the candidate reference genes.

10.7717/peerj.7133/supp-3 Supplemental Information 3 Primer efficiency based on standard graphs between target DNA dilutions vs. Ct values of fourteen reference genes.

10.7717/peerj.7133/supp-4 Supplemental Information 4 (A) average Ct values and (B) 2^-ΔCt^ values of fourteen candidate reference genes of Hypericum perforatum in all samples.

10.7717/peerj.7133/supp-5 Supplemental Information 5 M-values of the candidate reference genes calculated by GeNorm. (TS) Different tissues, (SG) developmental stage seedlings, (ST) stress-treated seedlings, and (TT) data from all experimental conditions combined. Samples with lowest M value show the most stable reference genes and highest M value represents least stable genes.

## Additional Information and Declarations

### DNA Deposition

The following information was supplied regarding the deposition of DNA sequences: The reference genes of *Hypericum perforatum* sequences described here are accessible via GenBank accession numbers MK054303, MK106356–MK106365.

### Data Availability

The following information was supplied regarding data availability: The raw data are available in Tables S3 and S4. The raw data shows the average Ct values, 2^(−∆Ct)^ values, and *M*-values of 14 candidate reference genes of *Hypericum perforatum* in all samples.
