# Supplementary figures and images for: Reference genes for qRT-PCR normalisation in different tissues, developmental stages, and stress conditions of Hypericum perforatum
Source: PeerJ. 2019 Jun 20;7:e7133. doi: 10.7717/peerj.7133 (PMC6589333; doi:10.7717/peerj.7133)

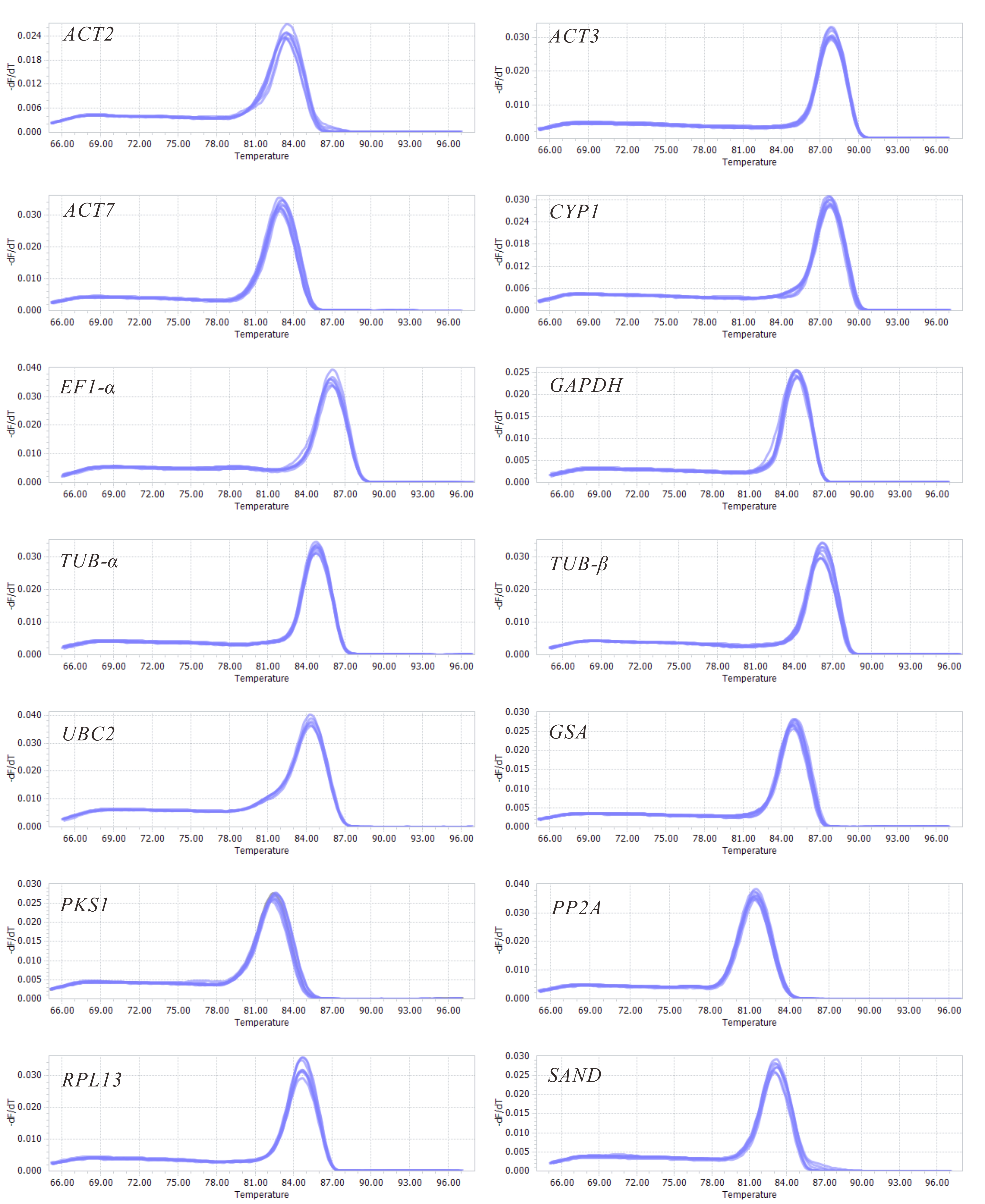

Supplement: Supplemental Information 1 — Melting curves for the fourteen candidate genes show single peaks. For each sub-graph, temperature is displayed in the x axis, the derivative reporter signal is displayed in the y axis. [file peerj-07-7133-s001.jpg]

(**TS**)


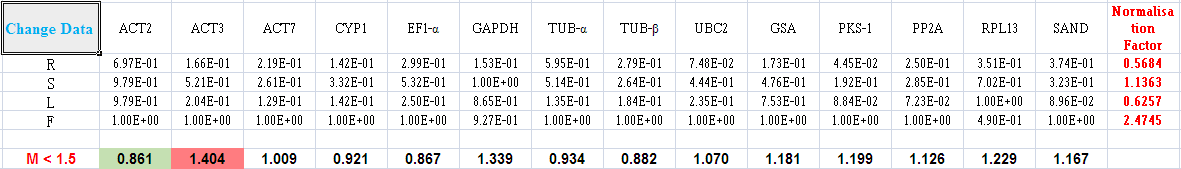


(**SG**)


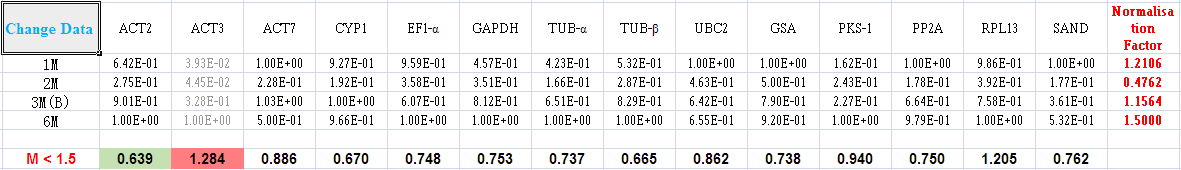


**(ST)**


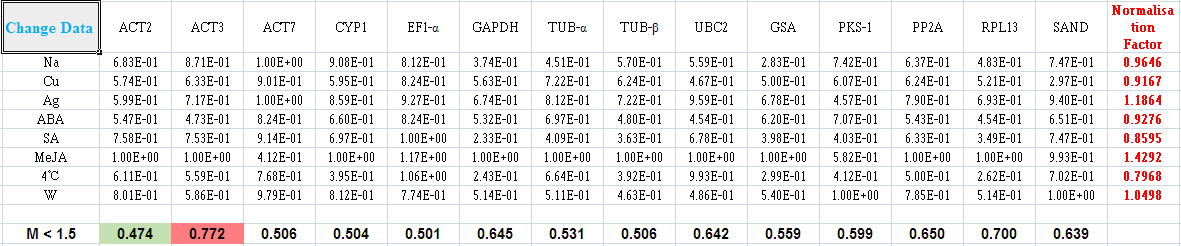


**(TT)**


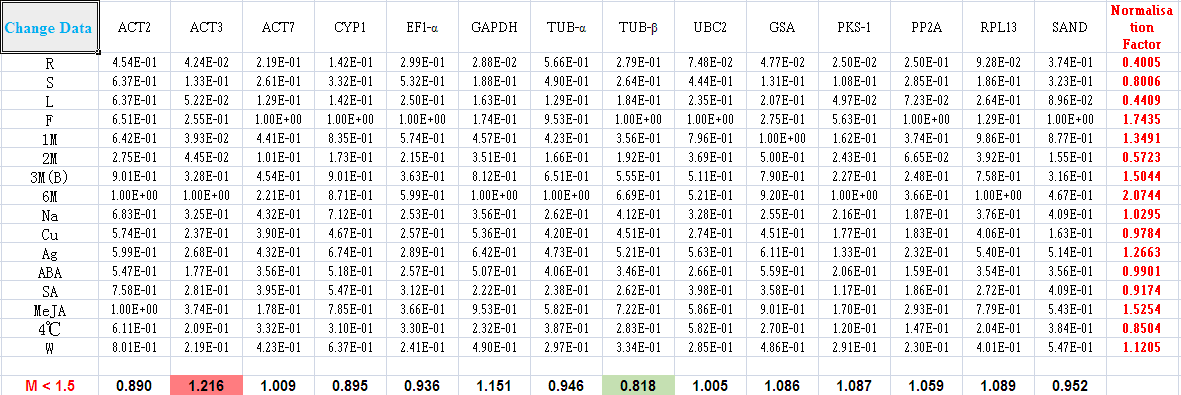

Supplement: Supplemental Information 5 — (TS) Different tissues, (SG) developmental stage seedlings, (ST) stress-treated seedlings, and (TT) data from all experimental conditions combined. Samples with lowest M value show the most stable reference genes and highest M value represents least stable genes. [file peerj-07-7133-s005.docx]
