# Supplementary material for: Reference genes for qRT-PCR normalisation in different tissues, developmental stages, and stress conditions of Hypericum perforatum
Source: PeerJ. 2019 Jun 20;7:e7133. doi: 10.7717/peerj.7133 (PMC6589333; doi:10.7717/peerj.7133)
Supplement: Supplemental Information 2 [file peerj-07-7133-s002.docx]

**>ACT2 (Hypericum perforatum, actin, complete cds)**

ATGGACAACCGAAACGTCGTCGTCTGCGACAACGGCACCGGATATGTCAAGTGTGGATTC

GCCGGCGAGAATTTCCCGACGTCGGTGTTCCCGTGCGTGGTCGGCCGGCCGATGCTCCGC

TACGAGGAGTCCCTCCACGACCACCAGCTCAAGGATATTGTGGTTGGAGAAAGTTGTGCG

GAGTTGAGGCACCAGCTGGATATATCCTACCCGGTACACAACGGCATTGTTCAGAACTGG

GATGACATGGGTCACGTTTGGGATCACGCCTTCTACAACGAGCTCAAAATTGACCCTTCA

GAGTGTAAGATTTTGCTGACTGATCCGCCCCTTAACCCCTCTAAAAATCGTGAAACAATG

GTTGAGACCATGTTCGAGAAGTACAACTTTTCTGGTGTCTTCATTCAAATCCAAGCTGTC

TTGACATTGTATGCCCAAGGTGGTTTGTTGACTGGATTGGTCATTGATTCTGGTGACGGT

GTCACTCATGTGGTTCCGGTTGTTGATGGATATTCATTTCCTCATCTTACTAAACGAATG

AATGTTGCTGGTCGGCACATCACATCCTATCTCATCGATTTACTTTCGAGGAGAGGTTAT

GCAATGAATAGGACATCTGACTTTGAGACTGTTAGAGAAATCAAAGAGAAGCTTTGCTAT

ATAAGCTATGATTATAAGAGGGAATACCAACTGGGACTTGATACCACCATTCTGGTGAAA

AATTACACTCTGCCAGATGGAAGGACCATCAAAGTAGGCACCGAGCGCTTTCAGGCCCCT

GAGGCTCTGTTTACACCAGAACTCATAGATGTTGAGGGTGATGGAATGGCTGACATGGTT

TTCCGTTGCATTCAGGAAATGGATATAGACAACAGGATGATGCTCTACCAGCACATTGTT

TTGAGTGGAGGGAGCACTATGTATCCTGGGTTACCTAGTCGGTTGGAGAAAGAAATACAA

GACCGGTATCTTGACACAGTTCTGAAGGGAAATAAGGATGGTTTAAAGAAATTGAGGCTG

CGGATTGAAGATCCCCCAAGAAGGAAGCATATGGTGTACCTTGGAGGTGCAGTTCTAGCG

GGAATTATGAAGGATGCACCTGAGTTTTGGATTAACAGAGACGATTATTTGGAAGAGGGA

CTTGCTTGCCTGAGCAAGTGTGGCCAGGCATGA

**>ACT3 (Hypericum perforatum, actin, complete cds)**

ATGGACCCCACTTCTCGACCCGCCGTCGTCATCGACAACGGCACCGGGTATACTAAGATG

GGATTCGCGGGGAATGTGGAGCCGTGTTTCATCCTTCCCACGGTGGTTGCGGTGAACGAG

TCTTTCCTCAACAACCAGTCACGGGGTTCGTCCAAGGCCAACTGGCTGGCCCAGCATAGC

GCGGGGGTCATGGCGGATCTCGACTTCTTTGTAGGCGACGAGGCGATTGCCAAGTCTAGA

TCTAGTAGTACTTACAACCTTAGCTACCCCATCCAGCATGGCCAGGTGGGTAATTGGGAC

TCCATGGAGAGGTACTGGCAGCAGTGTATATTTAATTACTTGCGGTGCGATCCCGAGGAT

CATTACTTTCTGCTCACCGAGAGCCCGCTCACTGCCCCGGAGAGTCGCGAGTATACTGGC

GAGATCATGTTCGAGACTTTCAATGTTCCTGGGCTATACATTGCTGTCAATTCGGTGCTT

GCTCTTGCAGCCGGGTACACCACATCCAAGTGCGAGATGACAGGAGTTGTGGTTGACATT

GGTGATGGGGCCAGTCATGTTGTGCCGGTTGCGGATGGCTATGTTATTGGTAGCAGTATC

AAGTCAATTCCTATCGCTGGGAAAGATGCGACTCTTTTCATCCAGCAGCTCATGAAGGAA

AGAGGAGAAAATGTTCCACCAGAAGATTCGTTTGAAGTAGCTAGGAAAGTGAAGGAAATG

CATAGCTATACTTGTTCGGACATTGTCAAGGAATTCAACAAACATGACAAAGAACCTTCC

AAGTATATCAAGCAATGGCGAGGTATCAAACCAAAAACTGGGGCTCCATACACTTGCGAT

ATTGGCTATGAACGATTTCTTGGCCCCGAGATTTTCTTTAGTCCTGAAATTTATAGCAGT

GACTTCACGACACCCTTGCCGGTTGTGATAGACAAATGTATTCAGTCTGCACCGATTGAT

ACAAGGAGGGCTTTATATAAGAACATAGTGTTGTCTGGTGGGTCAACTATGTTCAAGGAC

TTTGGCAGAAGGTTGCAACGAGATCTGAAGAAGATAGTGGACGCCCGTGTTCAATCATAC

GAGTCTAGACTTGTCGGAGAAGTAAAGTCACATCCACTGGAGGTCAATGTAGTCAGCCAT

CCTATTCAAAGATATGCAGTTTGGTTTGGGGGTTCTGTACTCGCCTCAACTCCCGAATTT

TTTGCGGCTTGTCATACAAAAGAAGAATATGACGAATACGGAGCAAGTATTTGCCGTACG

AATCCTGTTTTCAAGGGAATGTATTAG

**>ACT7 (Hypericum perforatum, actin, complete cds)**

ATGGACCCGGTGGTTATAGACGCGGGGTCAAGGCTTCTCAAGGCGGGCACCGCCGTCCCG

GAGCAGACCCCACCAGTGATAATTCCAACCCGAATGAAACGAATGGTCGAAGAAGAGCAG

CAGCAGGTCGACAATGCTTCGTTTGAGGAGGTCACTGTTGATCCTGTCGTTCGAGGTGTT

ATCAAAGATTGGGATGCTATGGAAGATCTGCTTCATTATGTTCTATACACTGGGCTCGAG

TGGGTTGAGGGAAACGAAGGTCAAATTTTGTTTACTGACCCACTCTCCACCCCTAAGGCT

GTCCGAGAACAGTTGGTGCAGCTGATGTTTGAAACATTTAACGTCTCCGGGTTTTATGCA

TCAGAGCAGGCCGTACTGTCGCTTTACGCAGTTGGTCGTATTTCAGGATGCACAGTTGAT

ATTGGTCATGGGAAGATAGATATCGCACCAGTACTTGAAGGTGCTGTTCAGCATATTGCC

TCCAGGAGATTTGAGATTGGTGGCATCGATTTGACTACCTTACTATCTGGAGAATTGAGT

AAATCTAATCCTCATGTCAATATCAGTATTTCTGATGCGGAAACCATAAAAGAGAGGTTT

GCTTGCTGTGCGGAAGACGAGGATGCCTATAGAAAGATCCAACACTTGAATGAGTCAGAA

GAACATACCCTACCTGATGGACAGGTGATAAGAATAGGAAGAGAAAGATATACTGTTGGT

GAAGCTTTATTCCAACCTTCCATTTTGGGCCTAGAAGCACATGGCATTGTAGAGCAGCTT

GTCCGCAGTATAACAACTGTTTCGTCCGAGCATCACCGTCAGCTGCTTGAAAACACTGTA

CTTTGCGGTGGAATAACTAAGATGCCTGGCTTTGAAGATAGGTTCCAGCGAGAAGCTCTC

TTATCCACATCATCTATTCGTCCATCTCTAGTAAAGCCTCCAGAATATATGCCAGACGGA

TTGACAGAATATTCAGCTTGGATTGGAGGTGCCATACTCGCCAAAGTCGTGTTTCCTCAA

AATCAACATTTAACCAAGGGAGAATACGACGAAACCGGTCCATCCATTGTCCATCGGAAG

TGCTTCTGA

**>CYP1 (Hypericum perforatum, cyclophilin, complete cds)**

ATGGCAAACCCTAGGGTTTTCTTCGACATGACCATCGGCGGGCAGCCGGCCGGTCGGATC

GTGATGGAGCTATACGCCGACGTCGTCCCCAAGACGGCGGAGAACTTCCGCGCCCTCTGC

ACTGGCGAGAAGGGAAAGGGAAGGAGCGGGAAGCCCCTCCACTACAAGGGATCCAGCTTC

CACCGTGTGATCCCCGACTTCATGTGCCAGGGCGGTGACTTCACCGCCGGGAACGGCACC

GGAGGAGAGTCCATCTACGGATCCAAGTTCGAGGACGAGAACTTCATCAAGAAGCACACC

GGCCCCGGGATCCTCTCCATGGCCAACGCCGGACCGGGGACCAACGGATCCCAATTCTTC

ATCTGCACCGCTAAGACCTCGTGGCTCGACGGCAAGCACGTCGTCTTCGGCCAGGTCGTC

GAGGGGCTCGACGTTGTCAAGGCGATCGAGAAGGTCGGATCCTCCTCCGGCCGCACGGC

AGGCCCGTTGTTGTGGCCGACTGCGGCCAGCTCTAG

**>****EF1-a (Hypericum perforatum, elongation factor 1-alpha, complete cds)**

ATGGGTAAGGAAAAGGTTCACATCAACATCGTGGTCATTGGACATGTGGACTCTGGCAAG

TCCACCACCACTGGTCACTTGATCTACAAGCTTGGAGGTATTGACAAGCGTGTGATTGAG

AGGTTCGAGAAGGAGGCTGCTGAGATGAACAAGAGGTCATTCAAGTATGCCTGGGTGCTT

GACAAGCTCAAGGCCGAGCGTGAGCGCGGTATTACCATTGATATTGCCTTGTGGAAGTTT

GAGACCACCAAGTACTACTGCACTGTCATCGATGCTCCTGGACATCGTGATTTCATCAAG

AACATGATTACTGGAACTTCCCAGGCTGATTGTGCTATCCTCATCATTGACTCGACAACT

GGTGGGTTTGAGGCTGGTATTTCCAAGGATGGGCAGACCCGTGAGCACGCCTTGCTTGCT

TTCACCCTTGGTGTCAAGCAAATGATTTGCTGCTGCAACAAGATGGATGCCACCACTCCC

AAGTACTCCAAGGCCAGGTACGAAGAAATTGTCAAGGAAGTGTCTTCCTACCTCAAGAAG

GTTGGCTACAACCCCGACAAAATCCCATTTGTTCCCATCTCCGGGTTTGAGGGTGACAAC

ATGATTGAGAGGTCTACCAACCTTGACTGGTACAAGGGACCAACCCTTCTTGATGCTCTT

GACATGATCAATGAGCCCAAGAGACCATCAGACAAGCCCCTTCGTCTCCCACTTCAGGAT

GTCTACAAGATTGGTGGTATTGGAACTGTGCCCGTGGGGCGTGTTGAGACAGGTGTTGTG

AAGCCAGGTATGGTTGTGACCTTTGGCCCTACTGGACTCACCACTGAAGTTAAGTCAGTT

GAGATGCACCACGAGGCTCTCTTGGAGGCTCTCCCTGGTGACAATGTGGGTTTCAATGTC

AAGAATGTTGCTGTGAAAGATCTCAAGCGTGGGTTTGTTGCATCCAACTCTAAGGATGAC

CCCGCCAAGGAGGCTGCCAACTTCACCTCCCAGGTCATCATCATGAACCACCCTGGCCAG

ATCGGCAACGGATATGCCCCTGTGCTTGATTGCCACACCTGCCACATTGCTGTCAAGTTT

GCTGAGATCCTGACCAAGATTGACAGGCGATCTGGTAAGGAGCTCGAGAAGGAGCCCAAG

TTTTTGAAGAATGGTGATGCAGGTATGGTTAAGATGATTCCCACCAAGCCCATGGTTGTG

GAGACCTTCTCCGAGTACCCCCCACTTGGTCGTTTTGCTGTGAGGGACATGCGCCAGACG

GTTGCTGTCGGTGTGATCAAGAGTGTTGAGAAGAAGGACCCAAGTGGGGCCAAGGTGACC

AAGTCCGCAGCAAAGAAGAAATGA

>**TUB-α (****Hypericum perforatum, TUB-alpha, complete cds)** ATGAGAGAAGTGATAAGCATACACATAGGGCAGGCGGGGATCCAGGTGGGGAATTCTTGC

TGGGAGCTTTACTGCCTCGAACATGGCATCCAGCCCGATGGCACCATGCCCAGTGATACT

ACTATAGGTACCGAGCACGATTCTTTCAACACCTTCTTCAGCGAAACTGGCTCCGGAAAG

CATGTCCCCAGAGCTATATTTGTTGATCTCGAGCCTACTGTCATTGACGAAGTTCGGACA

GGATCCTACAGGCAACTCTTCCACCCTGAGCAGCTTATTTCAGGCAAAGAAGATGCTGCT

AATAACTTTGCCAGAGGACATTACACTGTTGGCAGAGAAATTGTTGATCTTTGCCTTGAC

AGAGTGAGGAAGCTGGCTGATAATTGCACTGGATTGCAAGGGTTTTTGGTATTCAATGCT

GTTGGCGGCGGAACTGGTTCTGGTTTGGGTTCATTGCTCTTGGAACGCTTATCTGTGGAC

TATGGAAAGAAATCAAAACTTGGCTTCACCATATATCCATCTCCCCAGGTATCCACAGCT

GTCGTAGAACCGTACAATAGTGTGCTCTCCACTCATTCACTACTTGAGCACACAGATGTT

GCTGTGCTTCTTGACAACGAGGCCATCTATGATATATGCAGGAAATCCTTGGACATTGAA

AGGCCAACTTACACAAACTTAAATCGCTTAATATCACAAACAATTTCATCATTGACCACT

TCCTTGAGATTCGATGGTGCCATCAACGTGGATGTTACAGAGTTTCAGACTAACCTTGTC

CCTTATCCTCGCATCCATTTCATGCTGTCATCTTATGCTCCAGTAATCTCTGCTGCAAAG

GCTTATCATGAACAATTATCAGTCCCTGAGATCACCAGTGCAGTGTTTGAACCTTCAAGC

ATGATGGCAAAATGTGATCCAAGGCATGGGAAGTATATGGCTTGCTGCTTAATGTACCGT

GGAGATGTAGTCCCGAAGGATGTCAATGCTGCGGTTGCCACCATTAAAACCAAGAGGACA

GTACAGTTTGTTGATTGGTGCCCAACTGGGTTCAAGTGCGGGATCAATTACCAACCACCA

ACGGTGGTACCCAGTGGCGATCTTGCAAAGGTGCAGCGTGCGGTGTGCATGATTAGCAAC

AACACTGCAGTTGCTGAAGTTTTCTCTCGAATTGACCACAAATTCGATCTGATGTACTCT

AAGAGAGCATTCGTCCACTGGTACGTTGGCGAGGGCATGGAAGAGGGTGAGTTTTCAGAA

GCTCGTGAAGACCTCGCTGCTCTTGAGAAAGACTACGAGGAAGTTGGTGCAGAAGGTGTT

GAAGAGGACGATGAACCAGAGGACTATTGA

**>TUB-β (Hypericum perforatum, TUB-beta, complete cds)**

ATGCCGAGAGAGATAATAACACTGCAGGTAGGACAATGCGGGAACCAGATCGGGATGGAG

TTCTGGAAGCAGCTCTGCCTCGAGCACGGCATCAGCAAGGACGGCATCCTCGAGGACTTC

GCTACTCAGGGAGGGGATAGGAAGGACGTGTTCTTCTACCAAGCGGACGACCAGCATTAC

ATCCCGAGGGCGCTGCTCATCGATCTTGAGCCTCGCGTCATCAACGGCATCCAGAACAGC

GAGTACCGCAACCTCTACAACCACGAGAACGTCTTCCTCTCCGACCACGGCGGCGGCGCA

GGTAACAATTGGGCCAGCGGTTACCACCAGGGCCAGGGCGTTGAGGAGGACATCATGGAT

ATGATCGATAGGGAGGCCGACGGGAGTGACAGCCTTGAAGGCTTCGTTCTCTGCCACTCC

ATTGCTGGAGGGACCGGCTCAGGTATGGGCTCGTATCTGTTGGAAGCGCTGAACGATCGG

TATAGCAAGAAGCTGGTTCAGACGTATAGCGTGTTTCCGAACCAGATGGAGACGAGCGAT

GTGGTCGTCCAGCCTTACAACTCTCTCTTGACGCTGAAGAGACTGACGCTGAATGCTGAC

TGTGTGGTTGTTCTTGACAACACCGCCCTCACTAGGATTGCCGTGGAGCGCCTGCATTTG

TCTAATCCTACTTTTGCTCAGACAAACTCTTTGGTGTCGACTGTCATGTCTGCTAGCACT

ACCACATTGCGGTACCCTGGATACATGAACAATGACTTGGTTGGTCTTTTGGCCTCTCTG

ATTCCGACACCGAGGTGCCATTTTCTCATGACAGGGTACACGCCCCTTACTGTGGAACGC

CAGGCTAATGTCATTCGTAAAACTACTGTACTGGATGTTATGAGAAGACTGCTTCAGTCC

AAGAATATAATGGTATCCTCTTATGCACGAACCAAGGAAGCCAGTCAAGCAAAGTACATT

TCTATATTAAACATCATTCAAGGGGAAGTTGATCCGACTCAGGTTCATGAAAGCTTGCAA

AGGATTCGAGAAAGGAAGCTTGTTAACTTCATTGAATGGGGTCCAGCAAGCATACAGGTT

GCTCTGTCCAGGAAGTCTCCTTATGTTCAAACTGCTCACAGGGTGAGTGGTCTCATGTTA

GCAAGCCATACTAGTATACGACACCTCTTCAGCAGATGTTTGACCCAGTATGAGAAGTTA

AGAAGGAAGCAAGCTTTTCTTGACAATTACCGGAAGTTCCCGATGTTCGCTGATAATGAT

CTGTCCGAATTTGATGAGTCGAGGGATATAATTCAGAGTCTGGTTGATGAATACAAGGCT

TGTGAGTCACCAGACTACATCAAATGGGGAATGGAGGATCCAGACCACCTTTTAACAGGA

GAAGGCAATGCATCAGGATCAGTTGATCCTAACATTGCTGTTTGA

**>UBC2 (Hypericum perforatum, Ubiquitin-conjugating , complete cds)**

ATGAGCAAAATTCTCTCTCCTCTGTCCTCGTCAGCAGTGTATCAACTACAACTAGGAGGA

GGAGGAGGAGGAGGAGGCGCCTTTGAGAGAGTTTCCTCCGCTGCTCGTAATCAGGAGGAA

GAGGAGCGGTTGTCATTGGGAAGCTTTCAATTGAATGGACCGGCTCTTGCGATTGAGGAG

GGGGGATTTTTGCTGATCGGTCTGTGTTGCGGTTGTGTAGGGTTCAAGATGTCTACTCCA

GCCCGGAAGAGGCTGATGAGGGACTTCAAGAGGTTGCAGCAAGATCCTCCTGCGGTATCA

GCGGGGCTCCTCAGGACAGTAATATCATGCTTTGGAATGCTACCAGATGATACCCCGTGG

GATGGAGGTACGTTTAAGCTGACACTTCAGTTTACTGAAGATTATCCAAACAAGCCACCA

AATGTGCGGTTTGTTTCTCGCATGTTCCATCCAAACATTTATGCGGATGGAAGCATTTGC

CTGGATATCCTGCAAAATCAATGGAGTCCTATCTATGATGTTGCTGCCATACTTACGTCA

ATCCAGTCCTTGCTCTGCGACCCAAACCCCAATTCCCCTGCGAATTCAGAAGCTGCACGG

ATGTTTAGCGAAAACAAACGTGAATACAATAGGCGGGTGAGGGAGATTGTTGAGCAGAGT

TGGACTGCTGATTAA

**>PP2A (Hypericum perforatum,** **protein phosphatase 2A, complete cds)**

ATGGGCGCGTTGTCGTCCGATTCGGTCAGTGATCTGGACGAGCAGATCTCCCAGCTCACC

CAGTGCAAGCCGCTCTCCGAACCTCAGGTTAGGGGACTTTGTGACAAGGCGAAGGAGATA

CTAATGCAAGAAAGCAATGTTCAGCCTGTGAACAGCCCAGTTACGATATGTGGTGATATA

CACGGGCAGTTTCATGATCTTGCTGAGCTCTTTCGGATTGGTGGCAAGTGCCCAGACACC

AATTACTTATTTATGGGAGACTATGTGGATCGTGGCTACTATTCTGTTGAGACTGTTACG

CTGCTAGTGGCGCTGAAAGTGAGGTATCCACGGAGAATTACCATTCTTAGAGGAAACCAT

GAAAGTCGTCAGATTACACAGGTTTACGGATTCTATGATGAATGCCTTCGGAAATATGGC

AATGCTAATGTTTGGAAGATCTTCACGGATCTGTTCGACTATTTCCCATTGACTGCATTG

GTTGAATCCGAAATATTTTGTCTACACGGTGGATTGTCCCCGTCGATAGAAACCCTCGAT

AACATCCGCAACTTTGATCGTGTTCAAGAAGTCCCTCATGAAGGGGCCATGTGTGATTTG

TTATGGTCTGATCCTGATGACCGTTGTGGCTGGGGCATATCACCCCGTGGAGCGGGATAC

ACCTTTGGTCAGGACATATCCGAACAATTCAACCATACAAACGGATTAAAGTTGATTGCC

AGAGCACATCAACTTGTTATGGATGGTTTTAACTGGGCCCATGAACAAAAGGTTGTCACC

ATATTTAGTGCCCCTAATTATTGTTACCGTTGTGGAAACATGGCCTCTATACTGGAAGTC

GATGACTGCAAGGGTCACACGTTTATTCAGTTCGACCCAGCTCCAAGGAGGGGAGAACCC

GACGTCACACGCAGAACACCTGATTACTTTCTGTGA

**>RPL (Hypericum perforatum, ribosomal protein L, complete cds)**

ATGGCAGTCCTCTGTGCTTCCTCCACACCCTCTTCTACTTGTACTCCCATTTCCACTATT

TCCACTCCAAGAATCTCCTCCTCCTCCTCCTCCTCCTCTCTGCTCTTTCCCGCCCGCCCC

CTGTTCCCCACCAAGAAGCCCTACCTCCTCCAAGCTTGCTGCGAGAAGGTAGAGAAAGAC

AAGCACAGGTTCGTCTCCAAAGTGCCCCTCGACCAGCGCTGGATGTTCGAGGAATGCGAA

GTCGACGGACCCGACATTTGGAACAACACTTGGTACCCGAAGGCGAAGGACCACATCAAT

ACCGATAAGCAGTGGTATGTTGTGGACGCCACGGATAAGGTTCTTGGGAGGTTGGCTTCC

ACCATTGCCATATATATCCGTGGGAAGAACTTGGCCACTTTCACTCCCAGTGTAGACATG

GGGTCCTTCGTCATTGTGACAAATGCCGAGAAAGTTTATGTTTCTGGTAATAAGAGAACC

CAGAAGCTCTATAGGCGGCATTCCGGGAGGCCTGGTGGAATGACGGTGGAGACATTTGAT

CAACTACAGAACAGGATCCCAGAAAGAATAATTGAACATGCCGTTCGTGGGATGCTTCCT

AAAGGGAGACTTGGCAGACAATTATTCAATCACCTTAAGGTTTACAAGGGTCCAGAGCAT

CCGCACGAGGCTCAGAAACCCATAGATCTACCATTAACGGACAAGAGGATTCAGAAGGAG

TTAGATTGA

**>SAND (Hypericum perforatum, SAND family protein, complete cds)**

ATGCCCTCCGCCGGTGCAGACACGGACTCCTGCTCCTCGCCCGACCGCGGCCCGGGGCGG

GTGGAGGAGGAGCTCGAATCCCTCGCGCTCGCGGATGAACAGGACGCCGACTGCGAGGAA

GGGGGAAGCGAATGCCGGAGCAGAACGGGAACCAAAGCGCGGGGGAGGAGGAGGAGGAGT

GGAAGGGGAGGAACTCGGAGGTGGAGGCGGTTTCGAGCCCTAGCAGCAGCGGTTACGCCG

GGGAGAGAGGGAGCACCAGCAGCGGGAGTGCCACGTCGGCGGGGCTGGGAGAGATCGAGG

TGGAGGAGATGGATAGGATACGGGATCCGATGGCGGTGCGGTTCGGATCCGCAGGCGCCG

TGGGCTCCCCGGAAGCGCGACCACGACGAGGACGATGCGTCTATATCATGGAGGAAAAGG

AAGAAGCATTTTTTCATATTGAGCAACTCAGGCAAACCAATCTATTCCAGGTATGGAGAT

GAGCACAAGCTAGCTGGGTTTTCAGCCACGTTGCAAGCCATCATTTCCTTTGTGGAGAAC

GGGGGAGATCGTGTAAAATTGGTTAGGGCGGGAAAGCATCAGGTTGTGTTCCTTGTTAAA

GGGCCTATTTACCTTGTTTGCATCAGTTGTACAGAAGAACCTTATGAGTCATTACGAGGT

CAACTGGAGCTCATTTATGGTCAGATGATAGTGATTTTGACGAAGTCAGTGAACAGATGT

TTTGTCAAGAACCCGAAGTTTGATATGACACCATTGCTCGGAGGAACAGATGCTGTCTTC

TCCTCTCTCATTCACTCTTTCAGTTGGAATCCAGCCACATTTCTTCACGCATACACTTGT

CAGCCTCTTGCTTATGCAACAAGACAAGCTGCTGGGGCAATACTTCAAGATGTTGCAGAT

TCTGGTGTACTCTTTGCAATTTTGATGTGCAAACATAAAGTTATCAGTCTTGTAGGCGCC

CAGAAAGCTTCTCTGCATCCTGATGACATGCTTCTGCTATCCAACTTCATTATGTCATCA

GAATCATTTAGGACTTCTGAATCTTTCTCTCCAATATGCCTGCCAAGATATAATCCGATG

GCGTTTTTGTATGCATACGTTCATTATTTTGATGTTGACACTTACTTGATATTGCTGACT

ACAAGTTCCGATGCCTTTTTTCATCTCAAGGATTGCAGGATTCGTATTGAGTCTGTTCTC

GTCAAGTCCAATGTTCTTAGCGAAGTTCAGAGATCCTTAATAGACGGAGGAATGCATATT

GAGGAGTTGCCTGATGATCCGCTCCCTCGCACTGGGACAACTTCTCCTCTTTTGGGACCA

CACAAACAACCTACAGATTCTCCTGATAGATACAGGGAGACGTTTGTCGGCATGGGTGGT

CCTGCTGGTCTTTGGCATTTCATTTACCGCAGCATTTTTCTTGACCAATATGTATCTTCG

GAGTTTTCATCTCCTATTAATACTCCACAACAGCAAAAGAGGCAACATCTTTACTCCCTT

GTCTGA
