# Supplementary material for: Reference genes for qRT-PCR normalisation in different tissues, developmental stages, and stress conditions of Hypericum perforatum
Source: PeerJ. 2019 Jun 20;7:e7133. doi: 10.7717/peerj.7133 (PMC6589333; doi:10.7717/peerj.7133)
Supplement: Supplemental Information 3 [file peerj-07-7133-s003.docx]

| Genes | Equation | Slope | Efficiency | coefficient (R^2^ ) |
| --- | --- | --- | --- | --- |
| *ACT2* | y=-3.381x+25.76 | -3.379 | 97.6% | 0.9962 |
| *ACT3* | y=-3.255x+31.25 | -3.255 | 102.9% | 0.9864 |
| *ACT7* | y=-3.153x+30.77 | -3.153 | 107.6% | 0.9991 |
| *CYP1* | y=-3.416x+29.64 | -3.416 | 96.2% | 0.9990 |
| *EF1-α* | y=-3.195x+28.83 | -3.195 | 105.6% | 0.9902 |
| *GAPDH* | y=-3.205x+23.52 | -3.205 | 105.1% | 0.9956 |
| *TUB-α* | y=-3.113x+29.93 | -3.113 | 109.5% | 0.9895 |
| *TUB-β* | y=-3.131x+28.30 | -3.131 | 108.6% | 0.9993 |
| *UBC2* | y=-3.366x+31.48 | -3.366 | 98.2% | 0.9985 |
| *GSA* | y=-3.465x+32.57 | -3.465 | 94.4% | 0.9980 |
| *PKS1* | y=-3.364x+33.57 | -3.364 | 98.3% | 0.9889 |
| *PP2A* | y=-3.435x+32.61 | -3.435 | 95.5% | 0.9952 |
| *RPL13* | y=-3.513x+31.26 | -3.513 | 92.6% | 0.9986 |
| *SAND* | y=-3.393x+29.91 | -3.393 | 97.1% | 0.9885 |
