# Supplementary material for: Reference genes for qRT-PCR normalisation in different tissues, developmental stages, and stress conditions of Hypericum perforatum
Source: PeerJ. 2019 Jun 20;7:e7133. doi: 10.7717/peerj.7133 (PMC6589333; doi:10.7717/peerj.7133)
Supplement: Supplemental Information 4 [file peerj-07-7133-s004.docx]

| Reference Gene | Samples (Average Ct values) | | | | | | | | | | | | | | | |
| --- | --- | --- | --- | --- | --- | --- | --- | --- | --- | --- | --- | --- | --- | --- | --- | --- |
|  | Tissues(TS) | | | | Stage(SG) | | | | Stress(ST) | | | | | | | |
|  | Root | Stem | Leaf | Flower | 1M | 2M | 3M | 6M | Na | Cu | Ag | ABA | SA | MeJA | 4 °C | W |
| *ACT2* | 27.31 | 27.02 | 27.12 | 26.99 | 27.01 | 28.23 | 26.52 | 26.37 | 26.92 | 27.17 | 27.11 | 27.24 | 26.77 | 26.37 | 27.08 | 26.69 |
| *ACT3* | 28.08 | 26.43 | 27.78 | 25.49 | 28.19 | 28.01 | 25.13 | 23.52 | 25.14 | 25.60 | 25.42 | 26.02 | 25.35 | 24.94 | 25.78 | 25.71 |
| *ACT7* | 26.34 | 26.09 | 27.11 | 24.15 | 25.33 | 27.46 | 25.29 | 26.33 | 25.36 | 25.51 | 25.36 | 25.64 | 25.49 | 26.64 | 25.74 | 25.39 |
| *CYP1* | 20.98 | 19.75 | 20.98 | 18.16 | 18.42 | 20.69 | 18.31 | 18.36 | 18.65 | 19.26 | 18.73 | 19.11 | 19.03 | 18.51 | 19.85 | 18.81 |
| *EF1-α* | 26.11 | 25.28 | 26.37 | 24.37 | 25.17 | 26.59 | 25.83 | 25.11 | 26.35 | 26.33 | 26.16 | 26.33 | 26.05 | 25.82 | 25.97 | 26.42 |
| *GAPDH* | 23.27 | 20.56 | 20.77 | 20.67 | 19.28 | 19.66 | 18.45 | 18.15 | 19.64 | 19.05 | 18.79 | 19.13 | 20.32 | 18.22 | 20.26 | 19.18 |
| *TUB-α* | 22.38 | 22.59 | 24.52 | 21.63 | 22.80 | 24.15 | 22.18 | 21.56 | 23.49 | 22.81 | 22.64 | 22.86 | 23.63 | 22.34 | 22.93 | 23.31 |
| *TUB-β* | 26.17 | 26.25 | 26.77 | 24.33 | 25.82 | 26.71 | 25.18 | 24.91 | 25.61 | 25.48 | 25.27 | 25.86 | 26.26 | 24.80 | 26.15 | 25.91 |
| *UBC2* | 32.32 | 29.75 | 30.67 | 28.58 | 28.91 | 30.02 | 29.55 | 29.52 | 30.19 | 30.45 | 29.41 | 30.49 | 29.91 | 29.35 | 29.36 | 30.39 |
| *GSA* | 25.76 | 24.30 | 23.64 | 23.23 | 21.37 | 22.37 | 21.71 | 21.49 | 23.34 | 22.52 | 22.08 | 22.21 | 22.85 | 21.52 | 23.26 | 22.40 |
| *PKS1* | 26.53 | 24.42 | 25.54 | 22.04 | 23.84 | 23.25 | 23.35 | 21.21 | 23.42 | 23.71 | 24.12 | 23.49 | 24.30 | 23.77 | 24.27 | 22.99 |
| *PP2A* | 24.07 | 23.88 | 25.86 | 22.07 | 23.49 | 25.98 | 24.08 | 23.52 | 24.49 | 24.52 | 24.18 | 24.72 | 24.50 | 23.84 | 24.84 | 24.19 |
| *RPL13* | 25.45 | 24.45 | 23.94 | 24.97 | 22.04 | 23.37 | 22.42 | 22.02 | 23.43 | 23.32 | 22.91 | 23.52 | 23.90 | 22.38 | 24.31 | 23.34 |
| *SAND* | 25.20 | 25.41 | 27.26 | 23.78 | 23.97 | 26.47 | 25.44 | 24.88 | 25.07 | 26.41 | 24.74 | 25.27 | 25.07 | 24.66 | 25.16 | 24.65 |

| Reference Gene | Samples ($2^{-\Delta\mathrm{Ct}}$ values) | | | | | | | | | | | | | | | |
| --- | --- | --- | --- | --- | --- | --- | --- | --- | --- | --- | --- | --- | --- | --- | --- | --- |
|  | Tissues(TS) | | | | Stage(SG) | | | | Stress(ST) | | | | | | | |
|  | Root | Stem | Leaf | Flower | 1M | 2M | 3M | 6M | Na | Cu | Ag | ABA | SA | MeJA | 4 °C | W |
| *ACT2* | 0.454 | 0.637 | 0.637 | 0.651 | 0.642 | 0.275 | 0.901 | 1.000 | 0.683 | 0.574 | 0.599 | 0.547 | 0.758 | 1.000 | 0.611 | 0.801 |
| *ACT3* | 0.042 | 0.133 | 0.052 | 0.255 | 0.039 | 0.045 | 0.328 | 1.000 | 0.325 | 0.237 | 0.268 | 0.177 | 0.281 | 0.374 | 0.209 | 0.219 |
| *ACT7* | 0.219 | 0.261 | 0.129 | 1.000 | 0.441 | 0.101 | 0.454 | 0.221 | 0.432 | 0.390 | 0.432 | 0.356 | 0.395 | 0.178 | 0.332 | 0.423 |
| *CYP1* | 0.142 | 0.332 | 0.142 | 1.000 | 0.835 | 0.173 | 0.901 | 0.871 | 0.712 | 0.467 | 0.674 | 0.518 | 0.547 | 0.785 | 0.310 | 0.637 |
| *EF1-α* | 0.299 | 0.532 | 0.250 | 1.000 | 0.574 | 0.215 | 0.363 | 0.599 | 0.253 | 0.257 | 0.289 | 0.257 | 0.312 | 0.366 | 0.330 | 0.241 |
| *GAPDH* | 0.029 | 0.188 | 0.163 | 0.174 | 0.457 | 0.351 | 0.812 | 1.000 | 0.356 | 0.536 | 0.642 | 0.507 | 0.222 | 0.953 | 0.232 | 0.490 |
| *TUB-α* | 0.566 | 0.490 | 0.129 | 0.953 | 0.423 | 0.166 | 0.651 | 1.000 | 0.262 | 0.420 | 0.473 | 0.406 | 0.238 | 0.582 | 0.387 | 0.297 |
| *TUB-β* | 0.279 | 0.264 | 0.184 | 1.000 | 0.356 | 0.192 | 0.555 | 0.669 | 0.412 | 0.451 | 0.521 | 0.346 | 0.262 | 0.722 | 0.283 | 0.334 |
| *UBC2* | 0.075 | 0.444 | 0.235 | 1.000 | 0.796 | 0.369 | 0.511 | 0.521 | 0.328 | 0.274 | 0.563 | 0.266 | 0.398 | 0.586 | 0.582 | 0.285 |
| *GSA* | 0.048 | 0.131 | 0.207 | 0.275 | 1.000 | 0.500 | 0.790 | 0.920 | 0.255 | 0.451 | 0.611 | 0.559 | 0.358 | 0.901 | 0.270 | 0.486 |
| *PKS1* | 0.025 | 0.108 | 0.050 | 0.563 | 0.162 | 0.243 | 0.227 | 1.000 | 0.216 | 0.177 | 0.133 | 0.206 | 0.117 | 0.170 | 0.120 | 0.291 |
| *PP2A* | 0.250 | 0.285 | 0.072 | 1.000 | 0.374 | 0.067 | 0.248 | 0.366 | 0.187 | 0.183 | 0.232 | 0.159 | 0.186 | 0.293 | 0.147 | 0.230 |
| *RPL13* | 0.093 | 0.186 | 0.264 | 0.129 | 0.986 | 0.392 | 0.758 | 1.000 | 0.376 | 0.406 | 0.540 | 0.354 | 0.272 | 0.779 | 0.204 | 0.401 |
| *SAND* | 0.374 | 0.323 | 0.090 | 1.000 | 0.877 | 0.155 | 0.316 | 0.467 | 0.409 | 0.163 | 0.514 | 0.356 | 0.409 | 0.543 | 0.384 | 0.547 |

**(B)**
